# Cumulative live birth rates in women with endometriosis undergoing ART treatment

**DOI:** 10.1093/humrep/deaf191

**Published:** 2025-10-06

**Authors:** Repon C Paul, Rebecca Deans, Amanda Henry, Cecilia Ng, Ingrid Rowlands, Gita D Mishra, Jason Abbott, Georgina M Chambers

**Affiliations:** National Perinatal Epidemiology and Statistics Unit (NPESU), Centre for Big Data Research in Health (CBDRH), University of New South Wales, Sydney, Australia; School of Clinical Medicine, Medicine and Health, Discipline of Women’s Health, University of New South Wales, Sydney, Australia; School of Clinical Medicine, Medicine and Health, Discipline of Women’s Health, University of New South Wales, Sydney, Australia; The George Institute for Global Health, UNSW Medicine and Health, Sydney, Australia; School of Clinical Medicine, Medicine and Health, Discipline of Women’s Health, University of New South Wales, Sydney, Australia; Population Health Program, QIMR Berghofer Medical Research Institute, Brisbane, QLD, Australia; Australian Women and Girls’ Health Research Centre, School of Public Health, University of Queensland, Brisbane, Australia; Australian Women and Girls’ Health Research Centre, School of Public Health, University of Queensland, Brisbane, Australia; School of Clinical Medicine, Medicine and Health, Discipline of Women’s Health, University of New South Wales, Sydney, Australia; National Perinatal Epidemiology and Statistics Unit (NPESU), Centre for Big Data Research in Health (CBDRH), University of New South Wales, Sydney, Australia

**Keywords:** endometriosis, assisted reproductive technology, infertility, cumulative live birth rate, complete cycle

## Abstract

**STUDY QUESTION:**

How do cumulative live birth rates (CLBRs) in women with endometriosis compare to those with other infertility diagnoses undergoing ART?

**SUMMARY ANSWER:**

Women with endometriosis as the sole cause of infertility achieved higher CLBRs compared to those with additional infertility diagnoses (endometriosis-plus) or other non-endometriosis causes of infertility.

**WHAT IS KNOWN ALREADY:**

Endometriosis affects approximately 10% of women of reproductive age and is a major cause of infertility, with many women resorting to ART treatments in the hope of achieving a pregnancy. However, the comparative success rates of ART for these women, compared to those with other causes of infertility is not well understood.

**STUDY DESIGN, SIZE, DURATION:**

This retrospective cohort study included 79 318 women who initiated autologous ART between 2014 and 2019 in Australia and New Zealand, with follow-up through 2021 or the first live birth.

**PARTICIPANTS/MATERIALS, SETTING, METHODS:**

Participants were categorized into three groups based on infertility diagnosis: endometriosis-only (n = 4311), endometriosis-plus (n = 6312; endometriosis with other infertility factors) and other-infertility (n = 68 695; no endometriosis). Conservative and optimal CLBRs were calculated based on assumptions made about the chance of live birth for women who discontinued treatment.

**MAIN RESULTS AND THE ROLE OF CHANCE:**

Endometriosis was reported as the sole cause of infertility in 5% of women (endometriosis-only), while 8% had endometriosis with other diagnoses (endometriosis-plus). The remaining women had either other causes of infertility (63%) or unexplained infertility (24%). Depending on assumptions made regarding patients who discontinued treatment, the CLBR by the sixth complete cycle for women diagnosed with endometriosis-only ranged from 64% to 83%; for women with an endometriosis-plus diagnoses, the CLBR ranged from 54.3% to 68.7%; and for women without endometriosis, the CLBR ranged from 57.3% to 76.5%. Compared to women without endometriosis, the live birth rate was 6% higher in endometriosis-only group (RR: 1.06; 95% CI: 1.04–1.08) and 5% lower in endometriosis-plus group (RR: 0.95; 95% CI: 0.93–0.97). Compared to the endometriosis-only group, pregnancy loss was 46% higher (RR: 1.46; 95% CI: 1.35–1.59) in endometriosis-plus group.

**LIMITATIONS, REASONS FOR CAUTION:**

The study did not assess endometriosis severity or phenotype, which may influence ART outcomes.

**WIDER IMPLICATIONS OF THE FINDINGS:**

These findings provide critical data for counselling women with endometriosis regarding ART success. The higher CLBR in the endometriosis-only group suggests that isolated endometriosis does not negatively impact ART outcomes and highlights the need for tailored management in women with additional infertility factors.

**STUDY FUNDING/COMPETING INTEREST(S):**

This study is funded through the Medical Research Future Fund (MRFF) Research Data Infrastructure grant (MRFRFD000065). The sponsors had no role in the design and conduct of the study; data collection, management, analysis and interpretation; manuscript preparation, review, or approval; or the decision to submit for publication. FSANZ contracts National Perinatal Epidemiology and Statistics Unit (NPESU) of the University of New South Wales (UNSW) to prepare annual reports and benchmarking reports from the Australian and New Zealand Assisted Reproductive Technology Database (ANZARD): one of those datasets is used in this study. R.C.P. is a Research Fellow of the NPESU, UNSW. G.M.C. is the Director of the NPESU, UNSW. J.A. reports support from the Medical Research Future Fund and the Australian Government Department of Health and Aged Care, consulting fees from Hologic and Gedeon Richter, honoraria from Hologic, and advisory board participation with Gedeon Richter. G.D.M. reports funding from the NHMRC and the Australian Government Department of Health and Aged Care and is lead editor of a book published by Oxford University Press. C.N. reports institutional funding, an unpaid leadership role with ACTA and is a former employee of CSL Vifor. R.D., A.H., and I.R. declare no conflicts of interest.

**TRIAL REGISTRATION NUMBER:**

N/A.

## Introduction

Endometriosis affects approximately 10% of women of reproductive age (Rowlands *et al.*, 2021), with a significant proportion experiencing difficulties in conceiving (Barnhart *et al.*, 2002, Dunselman *et al.*, 2014). The pathogenesis of endometriosis-related infertility is complex and multifactorial often compounded by other factors that impact fertility such as age and parity. Several proposed mechanisms are thought to be involved, including pelvic anatomical distortion, altered peritoneal function, ovulatory problems, impaired implantation and the negative impact of ovarian endometriosis on sperm, oocytes and embryo quality, leading to attrition of early embryos and reduced implantation (Practice Committee of the American Society for Reproductive Medicine, 2012; Harb *et al.*, 2013; Fenner, 2024).

Women with endometriosis frequently undergo ART, such as IVF, to achieve pregnancy (Barnhart *et al.*, 2002). However, the impact of endometriosis on ART outcomes remains a topic of ongoing debate, with varied results. Several studies have reported that endometriosis negatively influences ART success rates, with reduced oocyte quality, lower fertilization and implantation rates, and ultimately lower live birth rates (Azem *et al.*, 1999; Barnhart *et al.*, 2002; Aboulghar *et al.*, 2003; Omland *et al.*, 2005; Kuroda *et al.*, 2009). Conversely, other studies describe comparable pregnancy and live birth rates between women with endometriosis and those with other infertility factors (Olivennes *et al.*, 1995; Stern *et al.*, 2013; Saraswat *et al.*, 2017).

Cumulative live birth rates (CLBRs) over several ART complete cycles (all fresh and thaw embryo transfers associated with one ovarian stimulation) undertaken by the same women offer a comprehensive measure of ART success, crucial for counselling patients with endometriosis, who may require multiple complete cycles to achieve a live birth. Despite its clinical significance, data on the impact of endometriosis on CLBR are limited and inconsistent (Feichtinger *et al.*, 2019; Boucret *et al.*, 2020). Additionally, little is known about how the presence of endometriosis combined with other infertility diagnoses influences ART success rates compared to women without a diagnosis of endometriosis.

This retrospective cohort study aims to address these gaps by estimating the CLBRs in women with endometriosis undergoing ART treatment, using data from the Australian and New Zealand Assisted Reproduction Database (ANZARD). By comparing women with endometriosis as their sole infertility diagnosis, those with endometriosis and additional diagnoses, and women without endometriosis, we aim to provide valuable insights into how endometriosis influences ART outcomes, particularly live birth rates.

## Materials and methods

### Study design and data source

This retrospective cohort study analysed data from ANZARD, an audited clinical quality registry that collects ART treatment and outcome data from all cycles performed in Australia and New Zealand (Newman *et al.*, 2021). It is a licencing requirement that fertility clinics submit their data to ANZARD and thus complete ascertainment is assumed (Paul *et al.*, 2020). The study included women who had their first autologous (patient’s own oocytes) ovarian stimulation cycle between 2014 and 2019. Records of all autologous thawed cycles were linked to their corresponding episodes of ovarian stimulation, allowing the identification of patient and treatment characteristics of woman at the time of ovarian stimulation. These women were followed-up through to the end of 2021 or until the first treatment-dependent live birth, with the follow-up period ranging from a minimum of 2 years to a maximum of 8 years. Cycles after the first live birth were excluded from the analysis.

### Infertility diagnosis and group classification

The ANZARD registry records the cause of infertility as specified by the treating clinician for each treatment cycle in the following non-mutually exclusive categories: tubal disease, endometriosis, other female factors, male factors and unexplained infertility. A woman or couple may have multiple infertility diagnoses either within a single treatment cycle or throughout the entire treatment period (2014–2021). For this study, women were categorized into three groups:

- *endometriosis-only* group comprised women whose sole infertility diagnosis was endometriosis throughout the treatment period;
- *endometriosis-plus* comprised women diagnosed with endometriosis along with at least one additional infertility diagnosis during any treatment cycle, comprising a diagnosis male infertility, tubal factor infertility or other female infertility factors;
- *Other-infertility* group comprised women without a diagnosis of endometriosis but with other infertility diagnoses, including male infertility, tubal infertility, other female factors or unexplained infertility.

Patients whose infertility diagnosis was recorded as ‘*Not recorded*’ across all infertility categories in all treatment cycles were excluded from the analysis.

### Outcome definitions

The primary outcome of interest was live birth, defined as the delivery of a live-born infant of at least 20 weeks gestation, or with a birth weight of at least 400 grams. A clinical pregnancy was defined as one that met at least one of the following criteria: ongoing at 20 weeks, ultrasound evidence of an intrauterine sac (with or without a foetal heartbeat), identification of chorionic villi in the products of conception, or confirmation of an ectopic pregnancy via laparoscopy or ultrasound. Pregnancy loss was categorized as a clinical pregnancy without a live birth.

### Statistical analysis

We compared the demographic and treatment characteristics across the three groups. Categorical variables were compared using Pearson Chi-squared test, while means of continuous variables across groups were compared using analysis of variance (ANOVA). Separate generalized linear models were used to assess how treatment outcomes—live birth, clinical pregnancy and pregnancy loss—differed in women with endometriosis compared to those without endometriosis. The models were adjusted for treatment year, maternal age and parity at the start of the first treatment cycle.

We calculated CLBRs of women up to six complete ART cycles. A complete ART cycle comprises all embryo transfers (including both fresh and thaw embryos) associated with one ovarian stimulation. Given the differing assumptions about the prognosis of women who discontinued ART treatment, we calculated two types of CLBRs: conservative and optimal. The conservative CLBR assumes that women who discontinued treatment would have zero probability of achieving a live birth if they had continued with ART treatment. This was calculated by dividing the number of women who achieved a live birth up to a specific cycle undertaken between 2014 and 2021 by the total number of women who had their first ovarian stimulation between 2014 and 2019. The 95% confidence intervals (CIs) of conservative CLBR were calculated using standard errors based on the binomial distribution. In contrast, the optimal CLBR assumes that women who discontinued treatment had the same probability of achieving a live birth as those who continued. It was estimated (with 95% CI) using the Kaplan–Meier method (Kaplan and Meier, 1958). Analyses were conducted in StataCorp. 2019 Stata Statistical Software: Release 16 (StataCorp LLC, College Station, TX, USA).

## Results

A total of 92 376 women underwent their first autologous fresh ART cycle between 2014 and 2019 in Australia and New Zealand. Of these, 13 058 (14.1%) were excluded from the analysis due to missing infertility data. Among the remaining 79 318 women with available infertility data, 4311 (5%) had endometriosis as the sole cause of infertility (*endometriosis-only*), 6312 (8%) had endometriosis combined with other infertility diagnoses (*endometriosis-plus*) and 68 695 (87%) had no endometriosis but other infertility (*other infertility*). In the endometriosis-plus group, 2068 women (33%) had more than one additional infertility factors (e.g. male factor, tubal factor or other female infertility factor) alongside endometriosis (Table 1).

**Table 1.** Causes of infertility among women undergoing their initial autologous ovarian stimulation cycle in Australia and New Zealand during 2014–2019 and followed until 2021.

Cause of infertility	Number of women (N = 79 318)	%
Endometriosis only	4311	5.4
Endometriosis plus other infertility (N = 6312)		
Endometriosis + Male factor	1390	1.8
Endometriosis + Tubal factor	746	0.9
Endometriosis + Other female infertility factors	2108	2.7
Endometriosis + two or more additional factors	2068	2.6
No endometriosis but other infertility (N = 68 695)		
Male-only	12 689	16.0
Tubal disease only	3341	4.2
Other female factor only	22 043	27.8
Unexplained infertility only	19 191	24.2
Tubal factor + Male factor	712	0.9
Tubal factor + Other female infertility factors	1450	1.8
Tubal factor + Male factor + Other female infertility factors	574	0.7
Male factor + Other female infertility factors	8695	11.0

### Demographic characteristics and treatment outcomes

The mean age of women at the time of the first ovarian stimulation cycle was slightly higher in the *other-infertility* group (34.7 years) compared to the *endometriosis-only* (33.9 years) and *endometriosis-plus* (34.1 years) groups (*P* < 0.001) (Table 2). The distribution of age groups also varied, with a higher percentage of women aged 40–44 in the *other-infertility* group (16.9%) compared to the *endometriosis-only* (10.2%) and *endometriosis-plus* (11.9%) groups.

**Table 2.** Demographic characteristics and treatment outcomes of women undergoing their initial autologous ovarian stimulation cycle in Australia and New Zealand during 2014–2019 and followed until 2021, classified by infertility diagnosis.

Characteristics/outcomes of treatment	Endometriosis-only (N = 4311)		Endometriosis-plus^1^ (N = 6312)		No endometriosis but other infertility^2^(N = 68 695)		*P*-value
	n	%	n	%	n	%	
Female age at first ovarian stimulation cycle, mean (SD)	33.9 (4.3)		34.1 (4.6)		34.7 (4.9)		**<0.001**
<30	657	15.2	1053	16.7	10 525	15.3	
30–34	1747	40.5	2278	36.1	22 803	33.2	
35–39	1456	33.8	2201	34.9	22 911	33.4	
40–44	440	10.2	751	11.9	11 595	16.9	
>44	11	0.3	29	0.5	861	1.3	
Previous pregnancy							**<0.001**
No	3961	91.9	5764	91.3	59 900	87.2	
Yes	350	8.1	548	8.7	8795	12.8	
Women with cancelled ovarian stimulation in the first stimulation cycle	292	6.8	529	8.4	6229	9.1	**<0.001**
Number of oocytes retrieved in the first ovarian stimulation, mean (SD)	8.9 (6.6)		8.7 (6.8)		9.2 (7.3)		**<0.001**
0–4	1140	26.4	1862	29.5	19 278	28.1	
5–9	1484	34.4	2062	32.7	21 290	31.0	
10–14	948	22.0	1291	20.5	14 784	21.5	
15–19	439	10.2	634	10.0	7495	10.9	
20 or more	300	7.0	463	7.3	5848	8.5	
Number of oocytes fertilized in the first ovarian stimulation, mean (SD)	5.2 (4.4)		4.9 (4.5)		5.1 (4.7)		**<0.001**
Year of first ovarian stimulation cycle							**<0.001**
2014–2015	1404	32.6	2181	34.6	23 409	34.1	
2016–2017	1267	29.4	2009	31.8	21 920	31.9	
2018–2019	1640	38.0	2122	33.6	23 366	34.0	
**Treatment outcomes**							
Number of women having pregnancy	3040	70.5	4166	66.0	44 650	65.0	**<0.001**
Number of women achieving live birth	2781	64.5	3527	55.9	39 734	57.8	**<0.001**

1 Endometriosis-plus = Endometriosis and one of the concomitant diagnosis, including male infertility, tubal disease, and other female factors.

2 No endometriosis but other infertility = No endometriosis but male infertility or tubal disease or other female factors or unexplained infertility.

Women in the *other-infertility* group had a higher rate of previous pregnancy (12.8%) compared to women with *endometriosis-only* (8.1%) or *endometriosis-plus* diagnosis (8.7%) (*P* < 0.001) (Table 2). Cycle cancellation rates (defined as ovarian stimulation initiated but not proceeding to egg retrieval) in the first ovarian stimulation cycle were also lowest in the *endometriosis-only* group (6.8%) and increased among women with *endometriosis-plus* (8.4%) and those with *other-infertility* diagnoses (9.1%) (*P* < 0.001). The mean number of oocytes retrieved was lower in women with *endometriosis-only* (mean = 8.9) and *endometriosis-plus* (mean = 8.7) compared to those with *other-infertility* diagnoses (mean = 9.2) (*P* < 0.001). The mean number of fertilized oocytes was 5.2 for *endometriosis-only* cases, slightly above the *endometriosis-plus* group (4.9) and comparable to *other-infertility* group (5.1; *P* < 0.001). Overall, women with endometriosis-only infertility achieved the highest pregnancy (70.5%) and live birth rates (64.5%) compared to the endometriosis-plus group (66.0% pregnancy, 55.9% live births) and other-infertility group (65.0% pregnancy, 57.8% live births) (*P* < 0.001) (Table 2).

### Associations between endometriosis and live births and pregnancy loss

After adjusting for maternal age, year of treatment, and parity, women with *endometriosis-only* infertility had a 6% higher likelihood of live birth compared to the *other-infertility* group (adjusted relative risk [aRR]: 1.06; 95% CI: 1.04–1.08) (Table 3). In contrast, women with *endometriosis-plus* diagnosis had a 5% lower likelihood of live birth (aRR: 0.95; 95% CI: 0.93–0.97) compared to the *other-infertility* group. Compared to *other-infertility* diagnoses, the risk of pregnancy loss was significantly lower in the *endometriosis-only* group (aRR: 0.88; 95% CI: 0.78–0.99) and significantly higher in the *endometriosis-plus* group (aRR: 1.46; 95% CI: 1.35–1.59).

**Table 3.** Association between endometriosis and pregnancy outcomes of women undergoing their initial autologous ovarian stimulation cycle in Australia and New Zealand during 2014–2019 and followed until 2021.

Fertility outcomes	No endometriosis but other infertility N (68 695)	Endometriosis-only (N = 4311); aRR* (95% CI)	Endometriosis-plus (N = 6312); aRR* (95% CI)
At least one clinical pregnancy	Ref	1.04 (1.02–1.05)	0.99 (0.98–1.01)
At least one pregnancy loss	Ref	0.88 (0.78–0.99)	1.46 (1.35–1.59)
Live birth	Ref	1.06 (1.04–1.08)	0.95 (0.93–0.97)

* Adjusted for year of treatment, maternal age and parity reported at the initial treatment cycle.

### Cumulative live birth rates

The CLBRs were calculated for up to six complete ART cycles (Table 4). Following the first complete cycle, the live birth rate was 39.6% for women with *endometriosis-only*, 27% for women with *endometriosis-plus* infertility, and 35.3% for those with *other-infertility* diagnosis. By the sixth cycle, women with *endometriosis-only* reached a higher CLBR (conservative 64.0%; optimal rate: 83.0%) compared to those with *endometriosis-plus* infertility (conservative 54.3%; optimal rate: 68.7%) and *other-infertility* (conservative 57.3%; optimal rate: 76.5%).

**Table 4.** Cycle-specific and cumulative live birth rates (complete cycle) for women who started their first autologous fresh cycle in Australia and New Zealand during 2014–2019 and followed until 2021 or the first treatment-dependent live birth.

Cycle number	Number of women starting cycle	Number of live births	No of Women who discontinued	Discontinuation rate	Cycle specific live birth rate	Conservative Cumulative live birth rate	Optimal cumulative live birth rate
**Endometriosis only**							

1	4311	1708	616	23.7%	39.6%	39.6%	39.6%
2	1987	608	389	28.2%	30.6%	53.7%	58.1%
3	990	243	212	28.4%	24.5%	59.4%	68.4%
4	535	115	127	30.2%	21.5%	62.0%	75.2%
5	293	61	72	31.0%	20.8%	63.4%	80.3%
6	160	22	43	31.2%	13.8%	64.0%	83.0%

**Endometriosis plus**							

1	6312	1704	647	14.0%	27.0%	27.0%	27.0%
2	3961	834	583	18.6%	21.1%	40.2%	42.4%
3	2544	436	485	23.0%	17.1%	47.1%	52.2%
4	1623	234	329	23.7%	14.4%	50.8%	59.1%
5	1060	142	229	24.9%	13.4%	53.1%	64.6%
6	689	79	161	26.4%	11.5%	54.3%	68.7%

**No endometriosis but other infertility**							

1	68 695	24 244	11 426	25.7%	35.3%	35.3%	35.3%
2	33 025	8894	7178	29.7%	26.9%	48.2%	52.7%
3	16 953	3592	4126	30.9%	21.2%	53.5%	62.7%
4	9235	1584	2423	31.7%	17.2%	55.8%	69.1%
5	5228	716	1438	31.9%	13.7%	56.8%	73.4%
6	3074	357	907	33.4%	11.6%	57.3%	76.5%

**Figure 1.**
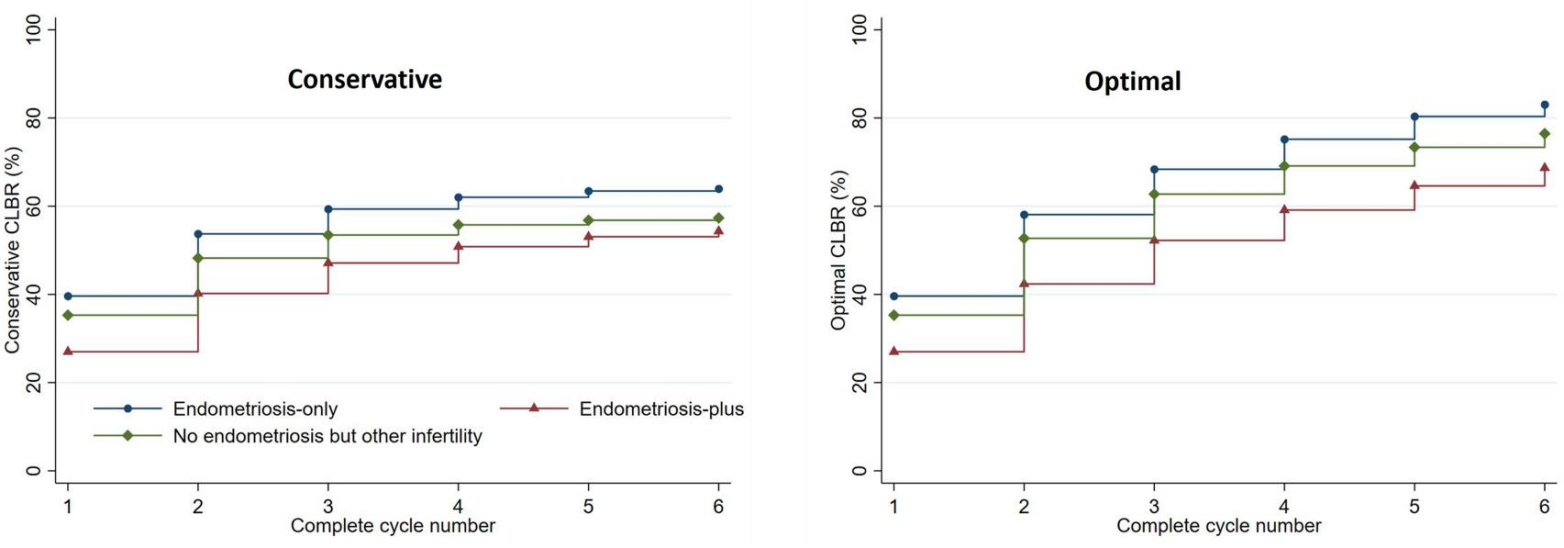
**Cumulative live birth rates (complete cycle) for women who started their first autologous fresh cycle in Australia and New Zealand during 2014–2019 and followed until 2021 or the first treatment-dependent live birth.** Conservative CLBR assumes women who discontinued had zero probability of live birth, while optimal CLBR assumes they had the same probability as those continuing ART. Endometriosis-plus group includes women diagnosed with endometriosis along with at least one additional infertility factor such as male, tubal, or other female causes.

ART success rates diminish with successive failed treatment attempts with the largest gains in CLBRs occur within the first two to three cycles across all groups. In the *endometriosis-only* group, conservative CLBR increases significantly from 39.6% to 53.7%, while the *endometriosis-plus* group rises from 27.0% to 40.2%, and the *other-infertility* group from 35.3% to 48.2% (Table 4; Fig. 1). As expected, gains beyond the second cycle are modest, with CLBRs increasing by 4–7% between the third and sixth cycle.

Discontinuation rates also progressively increased with each successive cycle across all three groups (Table 4). Among women with *endometriosis-only* infertility, rates rose from 23.7% after the first cycle to 31.2% by the sixth. The *endometriosis-plus* group exhibited the lowest rates, starting at 14.0% and reaching 26.4% by the sixth cycle. In contrast, the *other-infertility* group had the highest rates, peaking at 33.4% in the sixth cycle.

**Figure 2.**
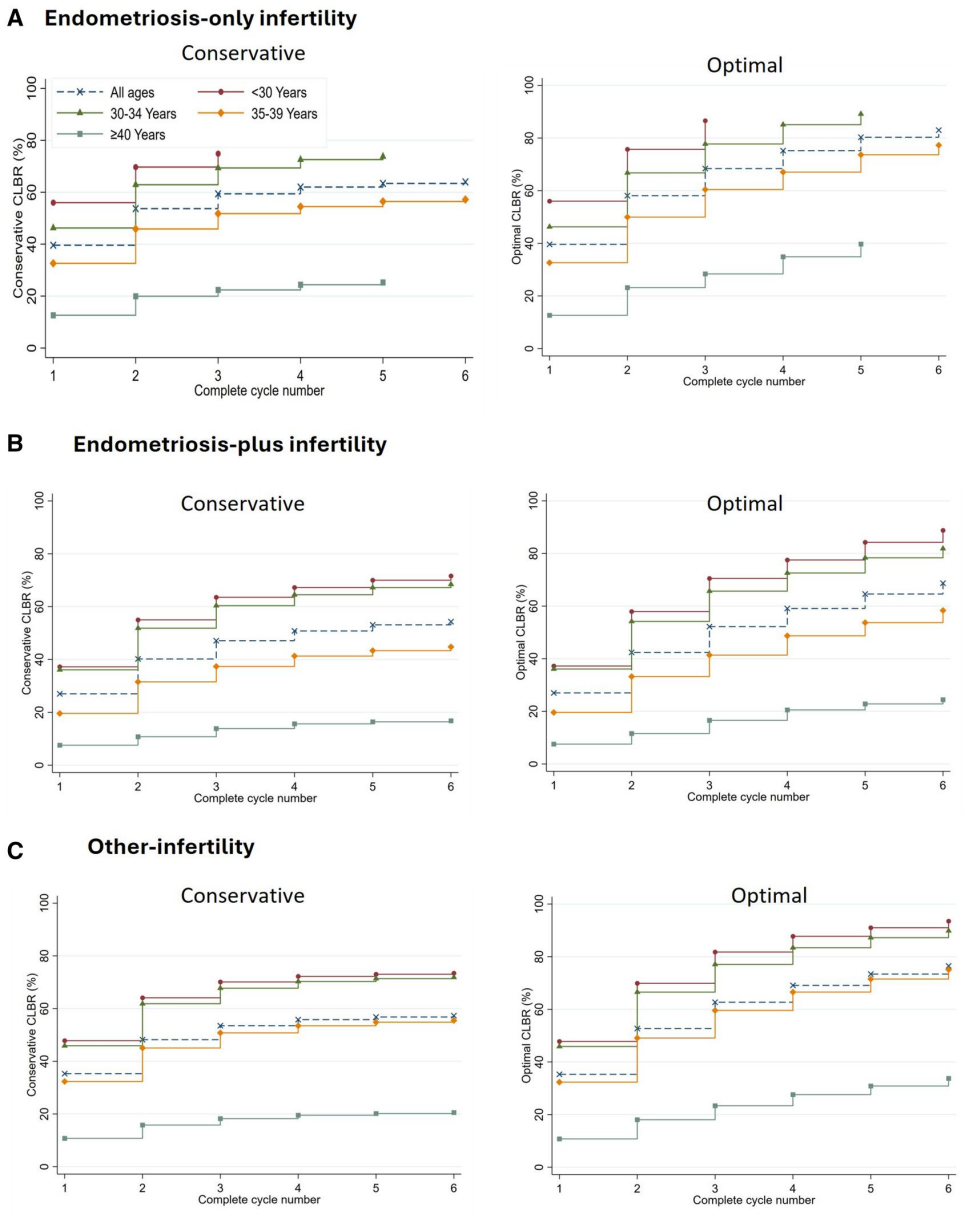
**Cumulative live birth rates by age group for women who initiated their first autologous fresh cycle in Australia and New Zealand during 2014–2019 and followed until 2021 or the first treatment-dependent live birth.** (**A**) Endometriosis-only infertility, (**B**) Endometriosis-plus infertility, (**C**) Other infertility. Conservative CLBR assumes women who discontinued had zero probability of live birth, while optimal CLBR assumes they had the same probability as those continuing ART. Endometriosis-plus group includes women diagnosed with endometriosis along with at least one additional infertility factor such as male, tubal, or other female causes.

The CLBRs by age group were calculated for women undergoing their first autologous fresh cycle (Fig. 2). For all age groups, CLBRs were consistently lowest in the *endometriosis-plus* group compared to the *endometriosis-only* and *other-infertility* groups. Across all infertility groups, live birth rates declined with advancing age. Women under 30 achieved the highest rates, with conservative CLBRs after three complete cycles reaching 74.9% for *endometriosis-only* (optimal rate: 86.6%), 63.5% for *endometriosis-plus* (optimal rate: 70.5%) and 70.1% for *other-infertility* groups (81.8%). Significant reductions were observed in women aged 35–39 and even more so in those aged 40 or older, with the most pronounced declines occurring in the *endometriosis-plus* group. Women over 44 years had negligible live birth rates, regardless of the infertility diagnosis.

## Discussion

This study provides a comprehensive age-stratified analysis of CLBRs in women with endometriosis undergoing ART treatment, highlighting the impact of endometriosis on treatment success. Our results demonstrated that women with endometriosis as their sole infertility diagnosis had higher live birth rates compared to women with endometriosis combined with additional infertility factors or other infertility diagnosis. Specifically, the adjusted relative risk for live birth was 6% higher in the *endometriosis-only* group compared to the *other-infertility* group (aRR: 1.06, 95% CI: 1.04–1.08), suggesting that isolated endometriosis does not adversely affect ART outcomes. Women with *endometriosis-plus* infertility had the poorest outcomes. After six complete ART cycles, women with *endometriosis-only* infertility achieved the highest CLBRs (64.0% conservative, 83.0% optimal), exceeding those with *endometriosis-plus* infertility (54.3%, 68.7%) and *other-infertility* (57.3%, 76.5%). These results are reassuring for patients with endometriosis and aid in counselling regarding ART success rates.

Our findings are consistent with previous studies reporting comparable or even improved ART outcomes in women undergoing ART with endometriosis as the sole infertility factor (Harb *et al.*, 2013; Senapati *et al.*, 2016; Maggiore *et al.*, 2024; Zhang *et al.*, 2025). The favourable outcomes in the *endometriosis-only* group are supported by growing evidence that endometriosis may not substantively compromise oocyte or embryo quality. Several studies have shown comparable fertilization, blastulation, implantation and euploidy rates between women with endometriosis and those with other infertility diagnoses (Juneau *et al.*, 2017; Ata and Telek, 2021; Bishop *et al.*, 2021; Benlioglu *et al.*, 2025). These findings support the hypothesis that oocyte competence may be maintained in women with endometriosis, particularly in the absence of other reproductive pathologies or advanced-stage disease.

Although the exact mechanisms of how endometriosis impacts reproductive outcomes are not yet fully understood (Macer and Taylor, 2012; Tanbo and Fedorcsak, 2017), it is known that endometriosis can lead to heightened pelvic inflammation, oxidative stress, and reduced gamete quality, potentially hindering the success of ART (Gupta *et al.*, 2006; Harb *et al.*, 2013). These effects are likely compounded in women with endometriosis who have additional infertility factors. For example, diminished ovarian reserve or male infertility significantly decrease the likelihood of conception (Harb *et al.*, 2013; Dunselman *et al.*, 2014; Tanbo and Fedorcsak, 2017). In our study, women with *endometriosis-plus* infertility had lower oocyte retrieval and fertilization rates than those with *endometriosis-only* or *other-infertility* diagnoses, suggesting the combined effects of inflammation, oxidative stress and gamete quality in multiple factor infertility are likely to contribute to these poorer outcomes. A population-based retrospective cohort study of 291 244 embryo transfer cycles reported higher cancellation rates among women with endometriosis and concomitant diagnosis (11.3%) compared to women with isolated endometriosis (8.5%) and women with other infertility diagnoses (8.9% for tubal infertility and 8.1% for unexplained infertility) (Senapati *et al.*, 2016), indicating a poorer prognosis. In our analysis, the cancellation rate for the *endometriosis-plus* group (8.4%) was significantly higher than in the *endometriosis-only* group (6.8%) but lower than in the *other-infertility* group (9.1%).

Few studies are available for direct comparison with our results that specifically estimate CLBRs among women with endometriosis after a defined number of complete ART cycles. Most prior research has calculated CLBRs either on a per-patient basis or per complete cycle, rather than following a specific number of complete cycles (Feichtinger *et al.*, 2019; Boucret *et al.*, 2020; Zhou *et al.*, 2022; Zimmermann *et al.*, 2023). However, these approaches do not provide insights into success probabilities after a specific number of completed ART cycles, a key area for patient-centred decision-making. Given that success rates are generally highest in the first cycle and progressively decline in subsequent cycles, having cycle-specific success data becomes critically important (McLernon *et al.*, 2016; Chambers *et al.*, 2017). Such detailed data not only provide a clearer picture of how outcomes evolve over time but also serve as a valuable tool for shared decision-making between clinicians and patients (Maheshwari *et al.*, 2015). It helps clinicians provide realistic guidance while empowering patients with cycle-specific success rates to make informed decisions, balancing parenthood aspirations with the emotional, physical and financial demands of continued treatment.

In our study, we reported two types of CLBR estimates—conservative and optimal. The conservative CLBR assumes that women who discontinued treatment had a zero probability of achieving a live birth if they had continued, although this assumption is overly pessimistic since not all patients who discontinue treatment would have zero chance of success. Conversely, the optimal estimate assumes that women who discontinue treatment have the same probability of live birth as those who continue, which is overly optimistic, as patients who discontinue often do so due to a poor prognosis. The range between these two estimates offers a reasonable assessment of the probability of live births after a specified number of complete cycles. This range allows clinicians to tailor their counselling to individual patients, particularly those with better prognosis (e.g. relative short period of infertility and no co-morbidities), who are more likely to approach the optimal estimates (Maheshwari *et al.*, 2015). While these population-based tables provide guidance on age-specific ART success rates for women with endometriosis, there are a number of unmeasured factors (e.g. BMI and duration of infertility) which influence ART success rates but are not explicitly included in this analysis. Furthermore, what is considered an acceptable ART success rate for patient and clinicians depends on a number of non-medical factors (e.g. cost, phycological burden) which should be considered to make shared decisions about fertility treatment options.

A key strength of this study is the large, complete, population-based dataset, which allows for robust comparisons of ART outcomes across multiple infertility diagnoses. However, there are limitations. First, we were unable to account for the severity and phenotype of endometriosis, which may influence ART outcomes. Advanced-stage endometriosis (Stage III/IV) is often associated with reduced ovarian reserve and impaired endometrial receptivity, leading to poorer outcomes, while mild cases (Stage I/II) may show comparable results to other single-factor infertility diagnoses (Kuivasaari *et al.*, 2005; Harb *et al.*, 2013). Furthermore, different endometriosis phenotypes, such as superficial peritoneal disease, deep infiltrating endometriosis or ovarian endometriomas, variably affect fertility and ART success (Vercellini *et al.*, 2023). Second, the diagnosis of endometriosis in this study was made by treating physicians in IVF clinics, based on clinical assessments and thus not uniformly made across patients. This diagnosis will often be reliant on a previous laparoscopy, imaging findings (such as endometrioma or deeply infiltrating disease) or clinical history. Endometriosis is often underdiagnosed or misdiagnosed, especially in the setting of infertility, due to its heterogeneous presentation, and reliance on clinical evaluation alone may lead to inconsistencies (Kuligowska *et al.*, 2005). Furthermore, a registry-based study cannot account for surgical variability in accurately diagnosing endometriosis or determining whether tubal disease may be attributable to endometriosis. Third, the *endometriosis-only* group in our study may reflect a subset of women with milder disease, as those with co-existing gynaecological conditions were classified into the *endometriosis-plus* group. Endometriosis is increasingly understood as part of a broader ‘uterine syndrome’, overlapping with conditions such as adenomyosis, fibroids and endometrial polyps (Brosens and Benagiano, 2011). Adenomyosis, in particular, is so closely linked to endometriosis that it is sometimes considered a related manifestation of the disease, with emerging evidence highlighting its role in the development of endometriosis-related symptoms, including pain and infertility (Vercellini *et al.*, 2023). However, due to the lack of data on these conditions in ANZARD, we were unable to classify women with overlapping pathology separately. As a result, the *endometriosis-only* group may represent a more favourable clinical profile, introducing the potential for selection bias and more favourable ART outcomes. This limitation should be considered when interpreting the relatively higher ART outcomes observed in this group. Fourth, in our study, the heterogeneity of *other-infertility* group, which includes a large proportion of women with 'other female factors’ (28%) and ‘unexplained infertility’ (24%) recorded in ANZARD, poses a challenge in interpreting comparative ART outcomes. The 'other female factors’ category likely encompasses diverse conditions including anovulation, diminished ovarian reserve and uterine abnormalities. Some of these conditions, particularly low ovarian reserve, are known to negatively influence ART outcomes (Busnelli *et al.*, 2021; Zhu *et al.*, 2024). As such, the apparently higher success rates in the endometriosis-only group may partly reflect poorer prognosis in parts of the *other-infertility* group rather than a truly superior outcome profile. This potential confounding should be considered when interpreting the relative differences in CLBRs. Finally, although statistically significant differences in certain baseline characteristics, such as maternal age, were observed across groups, these differences were generally small and may not be clinically meaningful. In large cohorts, even minor variations can reach statistical significance; therefore, such findings should be interpreted with caution.

This study provides valuable insights into ART success rates in women with endometriosis as a sole cause of infertility, those with endometriosis combined with other infertility factors, and those with infertility due to non-endometriosis causes. Women with isolated endometriosis achieved success rates comparable to those with other causes of infertility; however, women with endometriosis combined with additional infertility factors had lower success rates and higher pregnancy loss. Success rates declined significantly with advancing age and increasing numbers of ART cycles, with the highest success rates observed within the first three complete egg retrieval cycles. These findings are critical for patient counselling and highlight the importance of early intervention and tailored treatment strategies in women undergoing ART. Further research is needed to explore the role of endometriosis severity in influencing ART outcomes and to develop strategies for improving success rates in women with multiple infertility diagnoses.

## Data Availability

Data sharing agreement and ethics approval of this study prohibit the study team from making the dataset publicly available.
